# Simple, Economic, and Robust Rail-Based Setup for Super-Resolution Localization Microscopy

**DOI:** 10.1021/acs.jpca.3c01351

**Published:** 2023-05-10

**Authors:** Karim Almahayni, Gianluca Nestola, Malte Spiekermann, Leonhard Möckl

**Affiliations:** † Max Planck Institute for the Science of Light, Staudtstr. 2, 91058 Erlangen, Germany; ‡ Department of Physics, Friedrich-Alexander-University Erlangen-Nuremberg, 91054 Erlangen, Germany

## Abstract

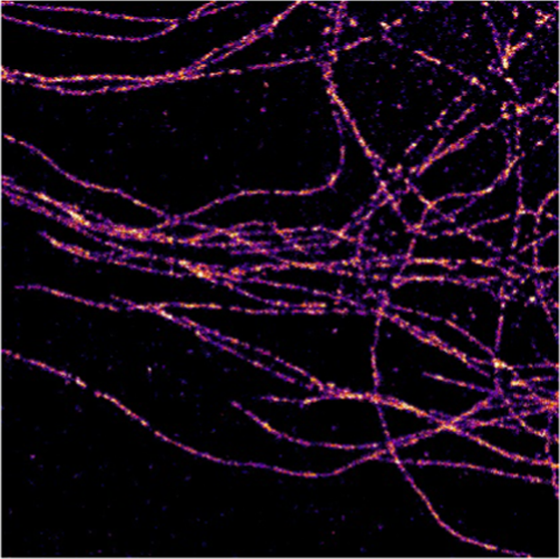

Research during the past 2 decades has showcased the power of single-molecule localization microscopy (SMLM) as a tool for exploring the nanoworld. However, SMLM systems are typically available in specialized laboratories and imaging facilities, owing to their expensiveness as well as complex assembly and alignment procedure. Here, we lay out the blueprint of a sturdy, rail-based, cost-efficient, multicolor SMLM setup that is easy to construct and align in service of simplifying the accessibility of SMLM. We characterize the optical properties of the design and assess its capabilities, robustness, and stability. The performance of the system is assayed using super-resolution imaging of biological samples. We believe that this design will make SMLM more affordable and broaden its availability.

## 1 Introduction

In biological systems, the nanoscale is full of tiny functional materials (e.g., chromatin, proteins, and glycans) that comprise the building blocks of life.^1^ Visualizing these molecules in detail and revealing their organization is critical for a comprehensive understanding of cellular processes. Light microscopy has proven to be a valuable tool to achieve this goal, mainly due to its capability to target a defined species inside a cell and due to its noninvasiveness.^2,3^ However, conventional light microscopy is subject to the diffraction limit, and consequently, resolving structures smaller than approx. 250 nm was not possible for a long time. Fortunately, recent years have seen the advent of super-resolution (SR) microscopy techniques. They enabled circumventing the diffraction limit, which allowed for resolutions down to the single-digit nanometer scale.^4−6^ These techniques unraveled the nanoscale architecture of a range of vital cellular complexes^7−9^ including endocytic vesicles,^10^ nuclear pore complex,^11^ apoptotic pores,^12^ glycocalyx,^13^ amyloid fibrils,^14^ immunological synapses,^15,16^ and neuronal cytoskeleton.^17^ The impact of SR techniques was highlighted by the Nobel Prize in Chemistry in 2014, awarded to Betzig, Moerner, and Hell.^18−20^

An important flavor of SR microscopy is single-molecule localization microscopy (SMLM). Normally, all fluorophores labeling a structure of interest emit at the same time. Thus, their diffraction-limited point spread functions (PSFs) overlap, yielding a blurred image. SMLM overcomes this inherent blurring by separating fluorophore activation in time in such a way that only a small subset of sparsely distributed fluorophores is active simultaneously.^21,22^ Experimentally, this can be, among other approaches, achieved using a high-power laser in conjunction with specialized chemical buffers. This transfers most fluorophores into the dark state (“shelfing”), leaving only a small subset active at the same time. The position of each of the well-separated single-molecule signals is determined by fitting with an appropriate mathematical model function (e.g., a two-dimensional, 2D Gaussian). This process is repeated over thousands of camera frames, yielding the super-resolved reconstruction from the accumulated single-molecule localizations.

In comparison to other SR methods, SMLM exploits relatively uncomplicated hardware. Therefore, SMLM has the potential for adoption as a routine microscopy system in biomedical research. The recipe for high-quality SMLM reconstructions is: (i) a sample where the structure of interest is labeled densely with bright, switchable fluorophores as poor sampling causes poor reconstructions,^23,24^ (ii) efficient detection, and (iii) precise and accurate localization. Of course, for accurate localization, proper software must be used.^25^ While the first component is a feature of the sample, the latter two are features of the microscope. From a technical perspective, efficient detection is related to a high numerical aperture (NA) objective for photon collection and a sensitive camera for detection. Finally, high localization precisions require a stable and robust setup, avoiding thermal drift, external vibrations, and misalignment. These key requirements comprise the anatomy of SMLM.

State-of-the-art SMLM setups are available both as custom builds and commercially. While powerful, they typically remain expensive, rather inflexible, and require skilled staff to operate and maintain. Thus, most SMLM setups exist in specialized laboratories or imaging facilities. In light of this, several recent efforts have focused on building systems that provide tailored, high-resolution, and cost-efficient solutions.^26−29^ Here, we expand on these efforts, introducing as a key innovation a rail-based design, inspired by previous work that used rail-based architectures for sensing applications where utmost precision as well as mechanical and thermal stability are mandatory, such as interferometry or measurement of diffusion coefficients.^30−32^ Setup designs described previously offer a variety of features, such as advanced illumination options like TIRF or HILO, three-dimensional (3D) imaging, or automated stage positioning. Our approach focuses on two key aspects: Minimizing cost and implementation of a rail-based system for maximal user-friendliness. Thus, as we are concerned with the characterization of the core functionality of our approach, the setup described here does not offer some of the features previous designs do. However, we note that the high flexibility of our approach enables the implementation of such expansions easily. For example, 3D imaging can be realized simply by adding a cylindrical lens to the emission path, and TIRF illumination is enabled by combining an objective suitable for TIRF with appropriate coupling of the excitation beam. Additionally, expansion of excitation options or implementation of specialized cameras is straightforward without the need to change the overall layout. Thus, we established a robust and low-cost layout with maximal user-friendliness, not compromising on data quality. The design can be set up, aligned, and operated by researchers with minimal background in experimental optics. We discuss the philosophy behind the design, outline its properties, and assess its capabilities. We hope that our approach will increase the accessibility of SMLM, setting the stage for its use as a routine system with outstanding resolution capabilities.

## 2 Materials and Methods

### 2.1 Preparation of Fluorescent Bead Samples

100 nm Tetraspeck or 200 nm flash red fluorescent beads (Thermo Fisher or Bangs Laboratories, respectively) were diluted (1:1000) in sterile-filtered water. 3 μL of the diluted suspension were pipetted onto 1.5 borosilicate coverslips (Thermo Fisher), distributed over an area with approx. 1 cm diameter, and left to dry in air. The slide was then mounted on a glass microscope slide (Thermo Fisher) using 3 μL of immersion oil (Leica Type F, Thorlabs). Finally, the coverslip was glued on the microscope slide using nail polish.

### 2.2 Tube Lens Alignment

To ensure adequate adjustment of the tube lens position, an experimental approach was chosen. 200 nm fluorescent beads were imaged at different tube lens positions. The experimental localization precision, defined as the standard deviations of the spread in the localization clouds, was determined. The tube lens was positioned where this spread was minimal, which coincided with the expected position at the focal point (Figure S1).

### 2.3 Pixel Size Measurements

The experimental image pixel size was determined by imaging the 1951 USAF Wheel Pattern resolution target (Thorlabs). The image pixel size was determined to be 88.5 nm.

### 2.4 Cell Culture

Human mammary epithelial cells (HMECs) were cultured in MCDB 131 medium (Thermo Fisher), supplemented with 10% fetal bovine serum, 10 ng/mL hEGF (both Thermo Fisher), 1% penicillin–streptomycin antibiotic cocktail, and 1 μg/mL hydrocortisone (both Sigma-Aldrich). Cells were seeded at a 1:4 ratio into eight-well chambered cover glasses (Thermo Fisher) and cultured for 48 h in a humidified 5% CO_2_ atmosphere.

### 2.5 Immunostaining

HMECs were fixed using 4% (w/v) paraformaldehyde (Thermo Fisher), permeabilized with 0.1% Triton-X-100 in phosphate-buffered saline with calcium and magnesium (PBS, Thermo Fisher), and blocked with 0.1% Tweeen-20 in PBS (PBST, Thermo Fisher) supplemented with 1% bovine serum albumin (BSA, Sigma-Aldrich) for 10 min. The cells were washed three times with PBS for 5 min between each of the steps. The cells were then incubated with 0.5 μg/mL mouse monoclonal anti-α Tubulin antibodies (Abcam) in PBST supplemented with 1% BSA for 1 h at room temperature. The cells were then washed three times with PBS for 5 min each. Then, the cells were incubated for 1 h with the secondary antibody. For one-color imaging, 2 μg/mL Goat Anti-Mouse IgG AF647 conjugate (Abcam) in PBST supplemented with 1% BSA was used. For two-color imaging, a cocktail of 2 μg/mL Goat Anti-Mouse IgG AF647 conjugate (Abcam) and 2 μg/mL Goat Anti-Mouse IgG CF568 conjugate (Sigma-Aldrich) in PBST supplemented with 1% BSA was used simultaneously. Finally, the cells were washed three times with PBS for 5 min each and stored in PBS at 4 °C in the dark.

### 2.6 Metabolic Labeling of the Glycocalyx and Copper-Catalyzed Click Reaction

HMECs were supplemented with 50 μM Ac_4_ManNAz (Thermo Fisher) in order to incorporate azido groups into sialic acid residues within the glycocalyx on the cell surface. The azido groups were subsequently conjugated with Alexa-647-alkyne (Thermo Fisher) via live-cell compatible copper-catalyzed click chemistry as previously described.^33^ Briefly, 2 days after seeding, the cells were washed three times with cold Dulbecco’s phosphate buffered saline (DPBS) on ice. The cells were then incubated with 50 mM CuSO_4_, 2.5 mM sodium ascorbate, 1 mM aminoguanidine, 250 mM tris-hydroxypropyltriazolylmethylamine (THPTA) (all Sigma-Aldrich), and 30 μM AlexaFluor647-alkyne in DPBS without Ca^2+^ and Mg^2+^ for 5 min at 4 °C in the dark. The cells were then washed three times with cold DPBS before fixation with 4% paraformaldehyde (Thermo Fisher) in DPBS for 20 min at room temperature.

### 2.7 2D One-Color and Two-Color SMLM

For SMLM, a reducing oxygen scavenging buffer that induces blinking of single fluorophores was employed according to the literature.^34^ The blinking buffer consisted of 2 μL/mL catalase (Sigma-Aldrich), 10% (w/v) glucose (BD Biosciences), 100 mM Tris-HCl (Thermo Fisher), 560 μg/mL glucose oxidase (Sigma-Aldrich), and 20 mM cysteamine (Sigma-Aldrich). The final pH is determined by the Tris-HCl buffer, which has a pH of 8. The PBS in which the fixed cells were stored was replaced by the blinking buffer. First, DL imaging was performed with low-intensity illumination of a few W/cm^2^. Then, the laser power was increased to approx. 3 kW/cm^2^. Image acquisition was started after a short delay in order to ensure that most fluorophores were shelved into a dark state. The exposure time was 50 ms, and 70,000–80,000 frames were obtained for microtubule imaging and approx. 40,000 frames for glycocalyx imaging, respectively. For two-color imaging, the same procedure was repeated using 561 nm excitation with approx. 3.7 kW/cm^2^. Histograms for signal and background photon counts, localization precision, and PSF widths are shown in Figure S2 (given as standard deviation).

### 2.8 Image Analysis

All images were processed using Fiji. SR reconstructions were obtained using ThunderSTORM.^35^ A 2D Gaussian was fitted to the detected single-molecule signals. The center of the Gaussian function was used as the position estimate for the *x*/*y* location of the single molecule with the uncertainty determined according to the method by Mortensen et al.^36^ Detailed detection and filtering conditions are reported in the Supporting Information (SI). Figures were prepared using Adobe Illustrator 2021. Graphs were plotted using Origin 2020.

### 2.9 3D Printing

All 3D-printed parts were printed using a fused deposition modeling printer (Ultimaker 3 Extended). Polylactic acid was chosen as a printing filament.

## 3 Results and Discussion

### 3.1 Design Philosophy

Our design philosophy relies on incorporating components that are reliable, economic, and commercially available, prioritizing high quality of the instrument and uncomplicated setup and alignment while maintaining cost efficiency and ensuring the ease of reproducibility (full parts list is provided Table S1). The cost of the final setup is approximately 10,000 € for a single-color design and 17,600 € for a multicolor design with a 561 nm laser. The increase in price for the multicolor option arises from the absence of affordable high-quality diode lasers in the 561 nm range, which is the excitation range for CF568, the second top-performing dye for SMLM alongside AF647. Nonetheless, both options are 6- to 10-fold cheaper than conventional setups, which often cost around 100,000 €.^21^ We note that our design is fully compatible with multiplexed PAINT, which, as demonstrated recently, allows for straightforward multicolor imaging with a single laser excitation wavelength.^37,38^ Furthermore, the flexibility of our approach allows for various extensions to meet specific requirements of a given research question.

**Figure 1:**
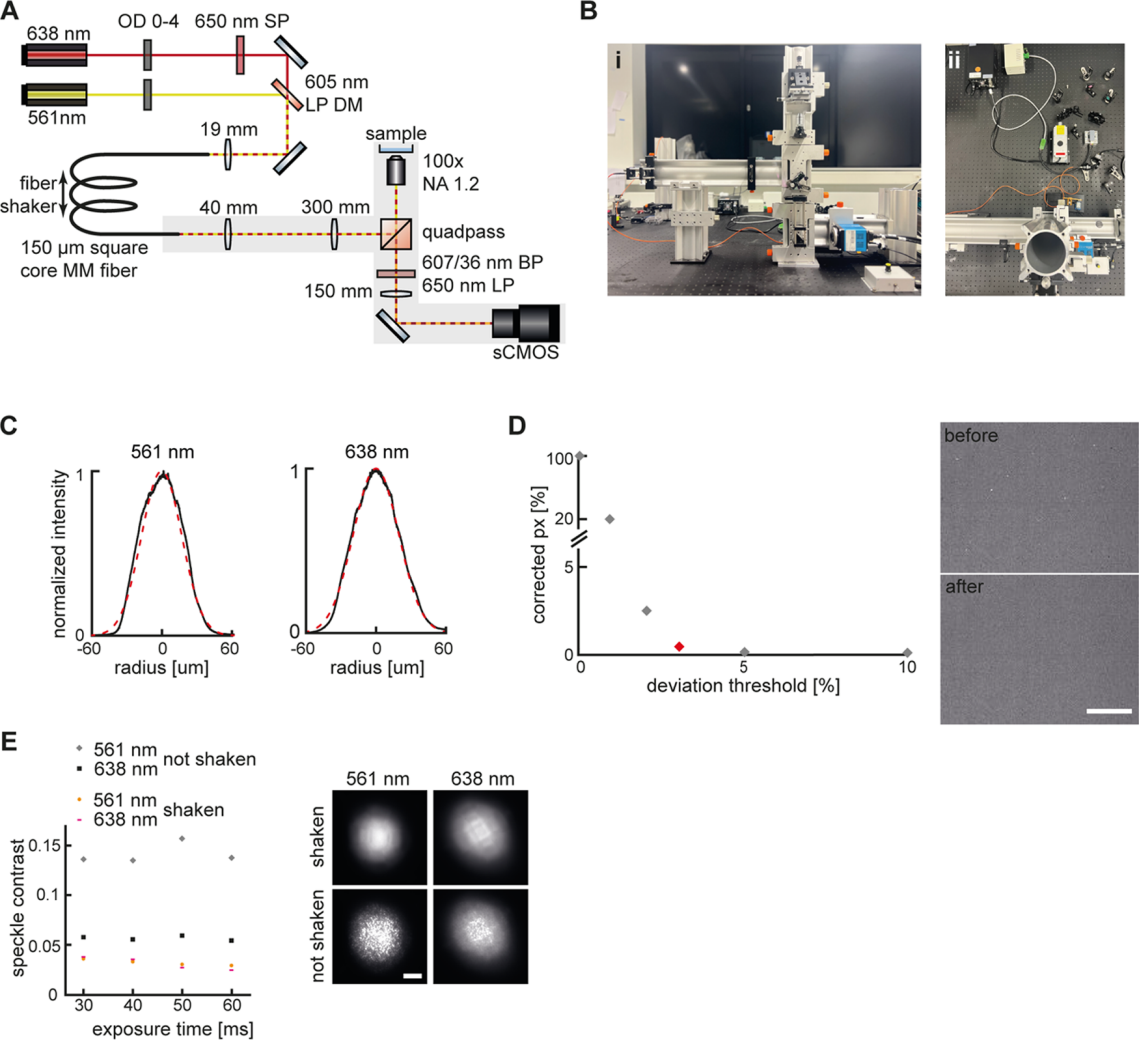
Setup design and characterization. (A) Setup design. Shaded areas indicate parts installed on rails. (B) Photographs of the setup (i) side view, (ii) top view. (C) Beam profiles for the 561 and 638 nm lasers. Black: Recorded profile, red: Gaussian fit. (D) Camera pixels before and after correction, chosen deviation threshold is indicated in red as 3%. (E) Reduction in speckle contrast with and without shaking the fiber for both excitation options. Scale bars: 3 μm (D), 20 μm (E).

The setup design and representative top- and side-view photographs are shown in Figure 1A,B. The setup integrates 561 and 638 nm lasers of a maximum power of 500 mW and 1 W, respectively (items 1 and 2 in Table S1). Cross sections through the beam profile of both lasers at the sample plane are shown in Figure 1C alongside Gaussian fits. Light generated by the lasers first passes through an optical density filter wheel (OD: 0–4; item 3) to enable attenuation of excitation intensity. The 638 nm is spectrally filtered using a 650 nm shortpass filter (item 4). The two laser beams are then united via a dichroic mirror with cutoff at 605 nm (item 5). An achromatic lens (*f* = 19 mm; item 6) is used to couple the lasers into a 150 × 150 μm square core multimode optical fiber (item 7). It is vital to highlight that the lasers used here are Class IV lasers which is the highest class. Intrabeam exposure leads to immediate and severe damages to the eye. Therefore, during laser alignment, wearing safety glasses, removing any reflective items, and ensuring restricted access to the room are essential. Furthermore, during imaging, the setup must be encased with suitable beam-blocking cardboards. The depictions of the unenclosed setup displayed in Figure 1B are only illustrative to ensure all parts are visible.

As discussed earlier, SMLM circumvents the diffraction limit by localizing the signal from individual fluorophores over time. Since prolonged imaging is required for sufficient sampling of the structure of interest, a sturdy and robust setup is critical for minimizing drift. Therefore, the core of the microscope was constructed on heavy metal rails (item 8) that ensure the sturdiness and robustness of the setup and mechanically couple all relevant parts, minimizing relative movements. Speckles generated inside the fiber are removed via mechanical agitation, which effectively suppresses unwanted patterns in the excitation profile (item 9; see Figures 1D, S3, and discussion below).

The beam is collimated using an achromatic lens with *f* = 40 mm (item 10) and focused onto the back focal plane of the microscope objective (100×, NA = 1.2, immersion oil; item 11) using a second lens with *f* = 300 mm (item 12). A quadpass dichroic filter is used to guide the excitation light to the objective and to separate emitted light from excitation light (item 13). After the quadpass, the emission light is further filtered dependent on the detected emission (607/36 nm BP for 561 nm excitation and 650 nm LP for 638 nm excitation, respectively; items 14 and 15). Finally, the tube lens (*f* = 150 mm; item 16) focuses the emitted light onto an scientific grade complementary metal oxide semiconductor (sCMOS) camera (item 17, quantum efficiency approx. 60% at 550–650 nm). Until recently, electron multiplying charge-coupled device (EMCCD) cameras were the gold standard for SMLM. However, scientific grade complementary metal oxide semiconductor (sCMOS) cameras have become an attractive alternative as they offer comparable noise characteristics and quantum yields at a lower cost.^39−42^ Even cheaper CMOS cameras are also available and have been used for SMLM before;^43^ however, these characterizations have also shown that the use of CMOS cameras yields lower localization precision and signal-to-noise ratio due to lower quantum yield and higher, irregular noise characteristics, potentially sacrificing reconstruction quality. Therefore, CMOS cameras were out of the decision matrix. Taking all of these factors into account, sCMOS was the camera type of choice for our approach. Again, aiming for maximal economic efficiency, we installed an affordable model of high-quality, air-cooled pco.edge 5.5 MP. To mount elements onto the heavy rails, carriers were used (item 18), and an xyz translation stage was implemented to move the sample in all three directions (item 19). To carry the sample, a custom sample carrier was engineered (Figure S3). Importantly, its design allows for firmly pressing down the sample using springs, minimizing drift. Finally, various standard optical parts, such as kinematic mounts, posts, clamping forks, plates, mirrors, mirror mounts, side clamps, a beam block, and screws, are required (items 20–31).

### 3.2 Microscope Characterization and Quality Checks

sCMOS cameras exhibit large sensors with millions of pixels. Compared to EMCCD cameras, one of the main drawbacks of sCMOS cameras are the noise characteristics, which are individual for each pixel. Therefore, careful correction of this pixel noise is mandatory to achieve highest resolution as highlighted previously.^39,44^ Broadly, there are two types of artifactual camera noises to be accounted for. First, individual pixels can exhibit deviating sensitivity (higher or lower), manifesting in the form of “hot” or “cold” pixels. Second, the offset of individual rows or columns of pixels can have different sensitivities, manifesting in stripe patterns. Both effects may introduce biases in the localization of single molecules and are thus determinantal for the final resolution.

Both effects are accounted for in our measurements. In order to identify hot and cold pixels, the first 4000 frames of a raw data stack were averaged. Due to the large time span, single-molecule signals in these frames average out, whereas hot and cold pixels, which are a systematic deviation, persist. These pixels were identified using a custom script. Briefly, each pixel is compared to its direct neighbors (8-connectivity). If the signal from the respective pixel was deviating more than 3% from the median of its eight neighbors, it was considered to be significantly brighter or darker, and a correction factor that sets the pixel to the median of its neighbors was recorded. Otherwise, the pixel was not considered to be significantly brighter or darker. We chose a conservative threshold in order to avoid over-corrections but, at the same time, ensured that no hot/cold pixels remained. The 3%-threshold was, in our measurements, ideal, as higher thresholds left deviating pixels uncorrected, whereas lower thresholds started to cause over-correction. This procedure yielded a pixel mask, which was then applied to the full stack of the raw data (Figure 1D). To correct for different sensitivities of whole columns of pixels, the no-light counts were recorded (i.e., the camera response in the absence of any light hitting the sensor). The resulting no-light count image was subtracted from each frame of the pixel-corrected raw data, yielding the final corrected raw data which was subsequently analyzed.

Furthermore, a homogeneous illumination profile is vital for SMLM since the photophysics of fluorophores depends on the intensity of the excitation light. The cost-efficient lasers used in our setup exhibit comparably poor beam profiles. As these are not ideal for SMLM, we employ a multimode fiber to easily clean up the beam profile. Unfortunately, the propagation through the fiber introduces speckles, arising from the propagation of several coherent laser modes inside the fiber, which would severely affect the quality of the final reconstructions if present in the excitation beam. A simple and straightforward method to reduce this phenomenon is mechanical agitation of the fiber, which we opted for in this design. The shaker constructed integrates a motor (3 V, max. 15,000 rpm), an electrical control, and three custom 3D-printed parts (Figure S3 and the Supporting Information). To verify successful despeckling, we employed a method previously described,^45^ which returns the “speckle contrast”, a metric that reports on the homogeneity of the excitation light (Figure 1E). As expected, the 561 nm laser exhibits stronger speckle patterns due to the longer coherence length of this laser compared to the 638 nm laser. However, with shaking, speckles are efficiently removed for both excitation wavelengths, and the speckle contrast is reduced to similar values of approx. 0.03.

### 3.3 Long-Term Stability and Robustness of the Setup

A core element of our design philosophy is to ensure high robustness of the system that can be operated by users with minimal experience in experimental optics. Therefore, we opted to put all relevant elements onto sturdy metal rails. Besides the increased mechanical stability, this approach also substantially simplifies the setup and alignment procedure: Instead of positioning elements in the three-dimensional space, two degrees of freedom that are typically not relevant for alignment are removed. Thus, the only variable is the position of the element along the optical axis, which is the one degree of freedom that matters for alignment. While aligning an optical element in three dimensions is a tedious task for a nonexpert, aligning it along a single axis is feasible.

**Figure 2:**
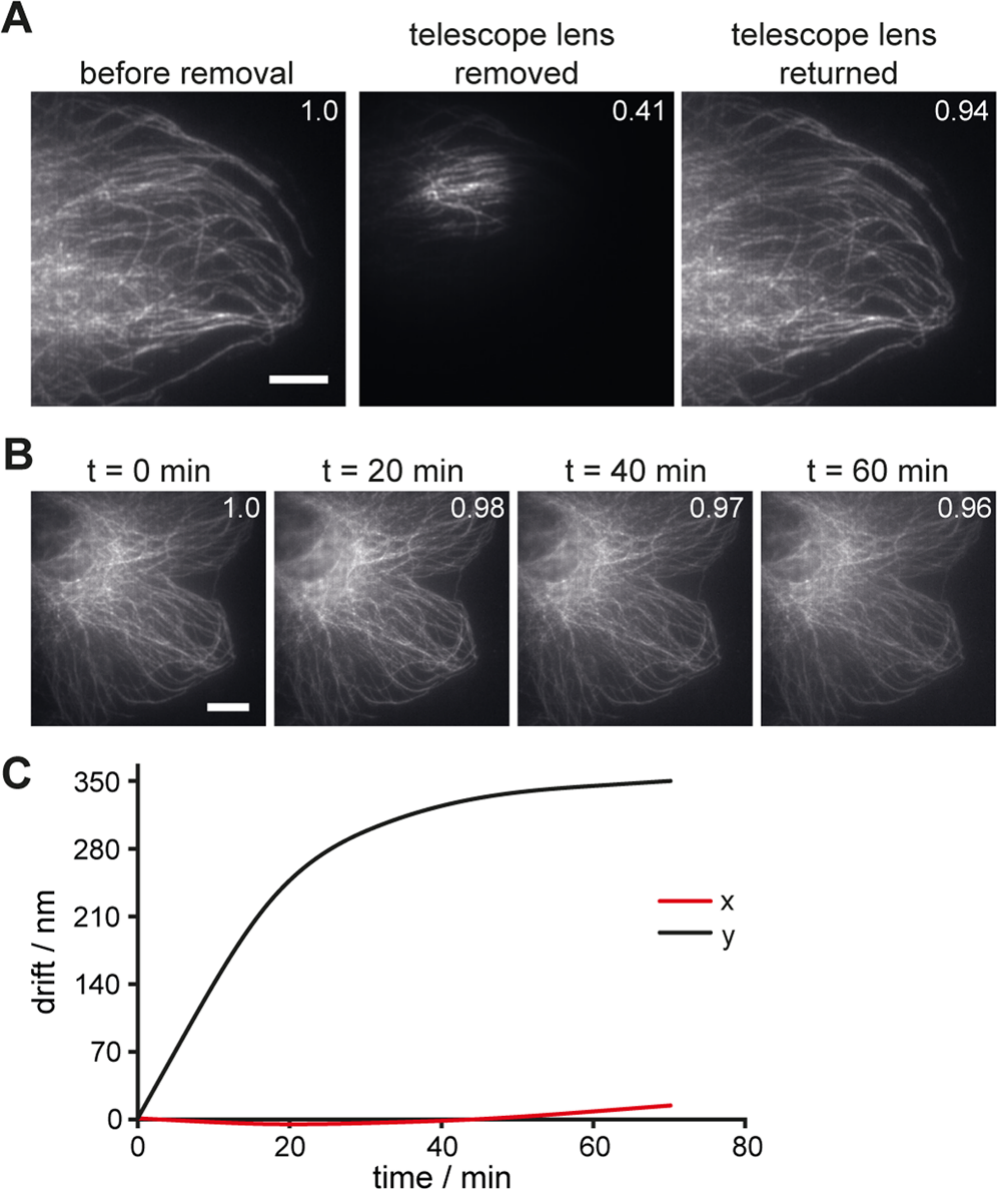
Setup robustness and stability. (A) Microtubule images acquired before and after the removal of the telescope lens. (B) Microtubule images acquired every 20 min for an hour. Pearson correlation coefficient is indicated at the top right of each image. The reference image is the first image for both sequences. (C) Nanometer drift of a single-molecule dataset determined by cross-correlation-based drift correction. Black: drift in *x*; red: drift in *y*. Scale bar: 10 μm. For additional data on robustness and mechanical stability, see Figures S4 and S5.

To verify the robustness of the design, we marked the position of one of the telescope lenses. Then, we acquired an image of immunostained microtubules, removed one of the telescope lenses, acquired a second image, and finally returned the lens and acquired a third image (Figures 2A and S4). The removal changed the excitation spot size as expected. Importantly, returning the lens recovered the original image. This is already evident by the appearance of the images. Moreover, the high Pearson correlation coefficient^46^ between the first and third images (but not between the first and the second image) confirms the visual impression.

Next, to assess the long-term stability of the microscope setup against drift, we acquired images of immunostained microtubules every 20 min over the course of 1 h, which is more than the typical acquisition time for an SMLM dataset (Figures 2B and S5). Again, these measurements confirmed the high stability of the setup as no visual drift was observed over the time investigated, which is also reflected in the high Pearson coefficient when comparing the images over time to the first image at *t* = 0. Additionally, we investigated the typically encountered nanometer drift using cross-correlation-based drift correction of the acquired one-color microtubule dataset (see below). The drift was minimal and, importantly, plateaued quickly, again highlighting the mechanical and thermal stability of the setup (Figure 2C).

### 3.4 Performance Indicators

**Figure 3:**
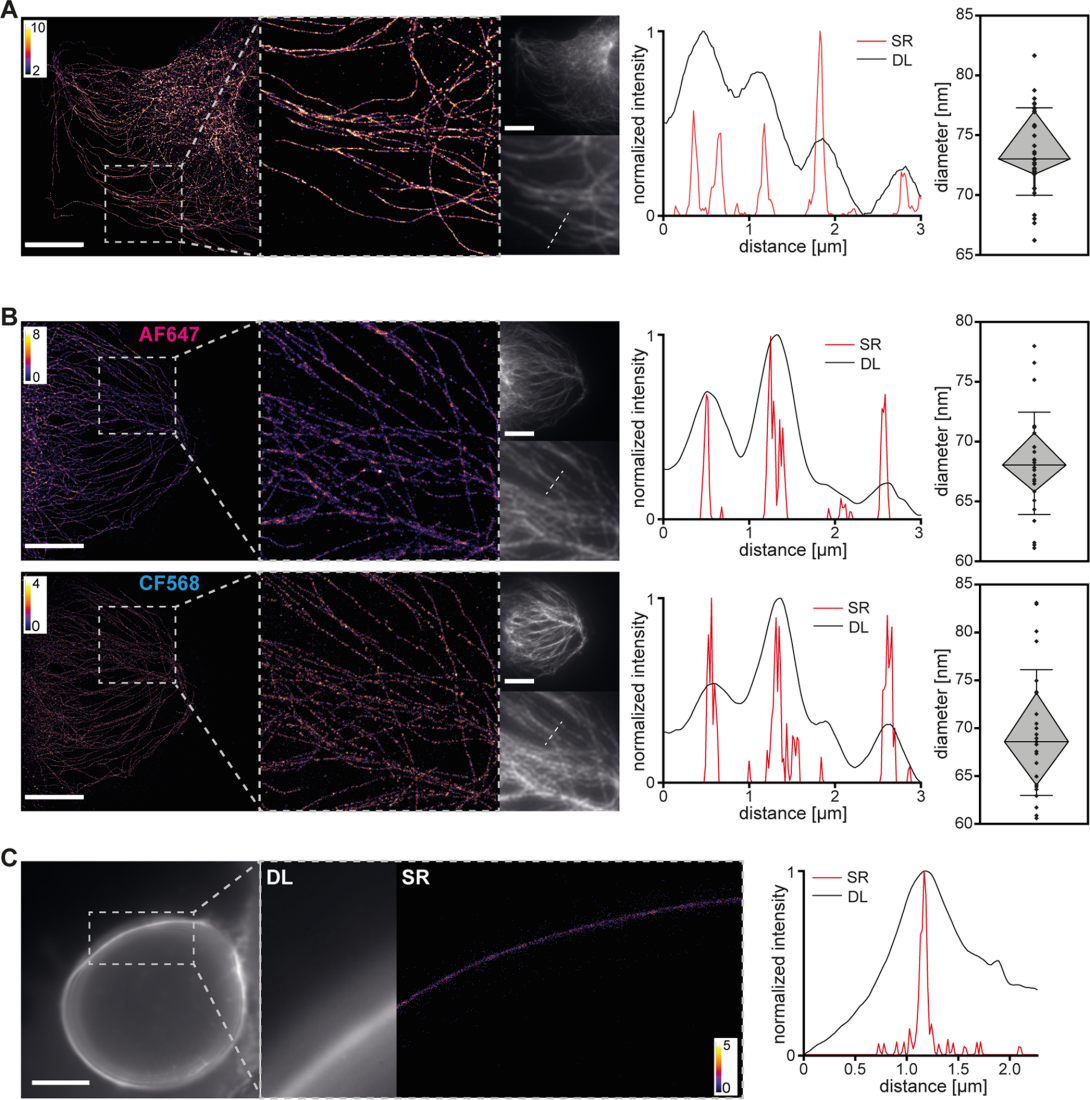
Super-resolution reconstructions of cellular structures in human cells. (A) One-color imaging and (B) two-color imaging of microtubules in HMECs. Line profiles correspond to white dashed lines in DL images. Red: SR, black: DL. SR reconstructions are shown as 2D histograms with 17.7 nm bin width. Microtubule widths are shown as diamond plots (diamond: second and third quartile, whiskers: standard deviation, median line, individual measurement values are scattered). *N* = 26–31 (C) One-color imaging of sialic acids on HMECs. Shown is a zoom-in of a membrane region perpendicular to the focal plane, with a direct comparison between DL image and SR reconstruction. A line profile perpendicular to the glycocalyx layer is shown on the right, both for the DL image (black) and the SR reconstruction (red). Scale bars: 10 μm.

To quantitatively assess the performance of the setup, we obtained 2D SR reconstructions of immunostained microtubules in one or two colors. Microtubules are a useful standard to assess the performance of SMLM setups as they are a clearly recognizable structure with well-defined dimensions. Microtubules in fixed HMECs were labeled either with AF647 for one-color imaging or with both AF647 and CF568 for two-color imaging. Acquisition of single-molecule signals and data analysis was performed as described above. We detected a few thousand photons per localization, as typical for the dyes used (for representative histograms of signal and background photons as well as localization precision and PSF width, see Figure S2, and for representative data showing single-molecule blinks, see Video S1). It should be noted that reconstructions obtained from imaging different dyes in multicolor SMLM may be affected by chromatic aberration, caused by slightly different focusing strengths of optical elements for different wavelengths. In addition, field-dependent aberrations might also be present. Such problems have been investigated in SMLM and other microscopy modalities in detail and can generally be corrected with high precision.^47−49^ As shown in Figure 3A/B, the SR reconstructions clearly exhibit much finer resolved structures compared to the diffraction-limited image, evident from the comparison between line profiles drawn over the same image region for the DL images and SR reconstructions. The expected width of an individual microtubule stained with a primary and secondary antibody is approx. 60–65 nm (25 nm tubule diameter plus 2 × 10 nm for a primary/secondary antibody combination on both sides).^50^ We randomly selected microtubules from the reconstructions, recorded the intensity profile across linear segments, and extracted the width via fitting with a Gaussian function. Our results are in line with the expected dimensions, both for one- and two-color imaging.

To further evaluate the performance of the setup using a more challenging sample, we imaged the glycocalyx: a complex meshwork of cell-surface glycans that are vital for various cellular processes (Figure 3C).^51^ Until recently, imaging the glycocalyx with high spatial resolution remained a challenging task owing to the lack of appropriate tools to specifically label the glycocalyx and due to its height, which is below the diffraction limit. Here, we combine metabolic labeling of sialic acid residues with super-resolution microscopy as described earlier.^13^ For one of the cells we imaged, the glycocalyx-covered membrane was sectioned perpendicularly, which allowed for direct extraction of the apparent thickness of the fluorescently labeled sialic acid layer. The dimensions were approximately 65 nm (width of a Gaussian fit to the SR line profile), which is consistent with our previous measurements^13^ and underscores the capability of the microscope design.

## 4 Conclusions

Accessibility to powerful microscopes suitable for SR imaging remains limited, in particular in nonspecialized laboratories and developing countries where studying disease-relevant biological systems below the diffraction limit remains hindered due to scarcity of funding. In this paper, we present a blueprint for a high-quality, easy-to-align, robust, and economic SMLM setup that enables straightforward multicolor SR imaging. We envision that future microscope designs might integrate 3D-printed,^52^ powerful phone cameras,^53^ LEGO assembled parts,^54^ and microfluidic chips,^55^ which will allow the construction of even simpler and more powerful microscopes. Overall, we believe that thanks to the continuing effort to increase the accessibility of SMLM systems, SR microscopy will become even more of a routine tool in the foreseeable future.

## Supporting Information Available

Detection and filtering settings, photon counts, PSF widths, localization precisions, 3D-printed parts, as well as setup design (PDF)
3D-printed and engineered parts and associated Solid Edge PAR files (ZIP)
Representative data showing single-molecule blinks (Video S1) (AVI)

## Supplementary Material

jp3c01351_si_002.zip

jp3c01351_si_003.avi

## Abbreviations

DL: diffraction-limited
PSF: point spread function
SMLM: single-molecule localization microscopy
SR: super-resolution
