# Supplementary material for: Simple, Economic, and Robust Rail-Based Setup for Super-Resolution Localization Microscopy
Source: J Phys Chem A. 2023 May 10;127(20):4553–60. doi: 10.1021/acs.jpca.3c01351 (PMC10226110; doi:10.1021/acs.jpca.3c01351)
Supplement: Supplementary file 1 — jp3c01351_si_001.pdf [file jp3c01351_si_001.pdf]

Electronic supplementary information for

"A Simple, Economic, and Robust Rail-Based Setup for Super-Resolution Localization Microscopy"

Karim Almahayni, Gianluca Nestola, Malte Spiekermann, and Leonhard Möckl

Max Planck Institute for the Science of Light, Staudtstr. 2, 91058 Erlangen, Germany

Table of contents

- Detection and filtering conditions for reconstructions
- Table S1: Parts list
- Figure S1: Tube lens positioning
- Figure S2: Signal and background photons, localization precision, PSF width
- Figure S3: Custom parts
- Figure S4: Setup robustness inspection via lens configuration alteration.
- Figure S5: Mechanical stability of the setup over 80 minutes.

Detection Settings
Each camera frame was filtered with a B-spline filter of order 3 and scale 2. Local maxima, corresponding to single-molecule signals, were detected with 8-neighborhood connectivity and a threshold of 1.1 or 1.2 times the standard deviation of the first wavelet level for microtubule and glycocalyx imaging, respectively. Detected local maxima were fitted with a 2D-Gaussian using least squares.

Filtering Conditions
- *One-color microtubule imaging, AF647*
  - Drift correction via cross correlation, 5 bins, 5x magnification
  - Merging: Maximal distance of 30 nm, maximally 5 off-frames, no limit of on-frames.
  - 100 nm < sigma < 300 nm
  - 6 nm < uncertainty < 28 nm
  - intensity < 15000 photons
- *Two-color microtubule imaging, AF647*
  - Drift correction via cross correlation, 5 bins, 5x magnification
  - Merging: Maximal distance of 30 nm, maximally 5 off-frames, no limit of on-frames.
  - 100 nm < sigma < 300 nm
  - 6 nm < uncertainty < 28 nm
  - intensity < 9450 photons
- *Two-color microtubule imaging, CF568*
  - Drift correction via cross correlation, 5 bins, 5x magnification
  - Merging: Maximal distance of 30 nm, maximally 5 off-frames, no limit of on-frames.
  - 90 nm < sigma < 190 nm
  - 7 nm < uncertainty < 29 nm
  - intensity < 8625 photons
- *One-color glycocalyx imaging, AF647*
  - Drift correction via cross correlation, 5 bins, 5x magnification
  - Merging: Maximal distance of 30 nm, maximally 10 off-frames, no limit of on-frames.
  - 40 nm < sigma < 300 nm
  - 8 nm < uncertainty < 30 nm
  - intensity < 12600 photons

Table S1: Parts list. Shaded rows are only needed for the two-color configuration.
All parts were obtained in 2021/22. Prices reflect the level of spring 2023.
Comparable components can also be obtained from other vendors.

Element ID	Element	Description	Supplier (Article number)	Price (€)
1	561 nm Laser	Maximum power 500 mW	Changchun New Industries (MGL-FN-561)	6,467.00
2	638 nm Laser	Diode Laser with a maximum power 1 W	Lasertack (PD-01382)	515.00
3	Filter wheel	Manual filter wheel mounts with neutral density filters	Thorlabs (FW1AND)	271.49 x2
4	Shortpass filter	Cut on wavelength: 650 nm	Thorlabs (FESH0650)	112.30
5	Dichroic mirror	Cut on wavelength: 605 nm	Thorlabs (DMLP605)	152.94
6	Achromatic lens	*f*=19 mm	Thorlabs (AC127-019-A)	46.16
7	Multimode fiber	Square core multimode optical fiber 150x150 μm	Thorlabs (M101L02)	86.07
8	Rails	3X 250 mm; 1X500 mm; 1X750 mm	Qioptiq Profile X95	496.00
9	Fiber shaker motor	For despeckling	Zhiqin 3-6V DC	8.00
10	Lens	Achromat, *f*=40 mm	Qioptiq (VIS ARB2; D=12.5, F=40)	129.00
11	Objective	100x, NA=1.2	Wetzlar	450.00
12	Lens	Achromat, *f*=300 mm	Qioptiq (VIS ARB2; D=25.4, F=300)	44.00
13	Quadpass	Quadline R405/488/561/635	AHF analysentechnic (F47-700)	577.00
14	Bandpass filter	Wavelength 607; bandwidth 36nm	Edmund Optics (84-102)	281.81
15	Longpass filter	Cut on wavelength: 650 nm	Thorlabs (FELH0650)	145.70
16	Tube lens	*f*=150 mm	Qioptiq (VIS ARB2; D=25.4, F=150)	44.00
17	Camera	sCMOS (Pico edge)	pco.edge 5.5 MP air cooled	4,000.00
18	Carriers	2X cross carrier<br>1X clamping carrier<br>4X carriers w/o holes<br>1X carrier with holes	Qioptiq (G026424000; G026408000; G026413000; G026412000)	676.00
19	xyz translation stage	12.7 mm XYZ Translation Stage	Thorlabs (MT3/M)	978.18
20	Kinematic optic mount	Three adjusters	Thorlabs (KS1)	77.37 x5
21	Posts	To hold elements	Thorlabs (RS1P4M, three pack)	59.85 x2
22	Clamping fork	15.9 mm Long Slot for M6 cap screw	Thorlabs (MSC2)	13.98 x5
23	Sliding filter mount	For emission filter	Thorlabs (CFS1)	161.97
24	Mirrors	Broadband Dielectric	Thorlabs (BB1-E02)	73.83 x3
25	Mirror Mounts	Kinematic	Thorlabs (KM-100R)	65.15 x3
26	Side Clamp	For quadpass	Newport (M1-RQ)	70.30
27	Pelical Mount	Kinematic	Thorlabs (KM100BP)	215.82
28	Adapter plate	For fiber	Thorlabs (SM1FC2)	30.70
29	Holder Plates		Qioptiq (G065061000)	34.00 x2
30	Beam Block		Thorlabs (LB1)	52.40
31	Small parts	Screws, hex keys, springs etc.		200.00
One color price estimate: approx. 10,000 €<br>Two color price estimate: approx. 17,600 €				

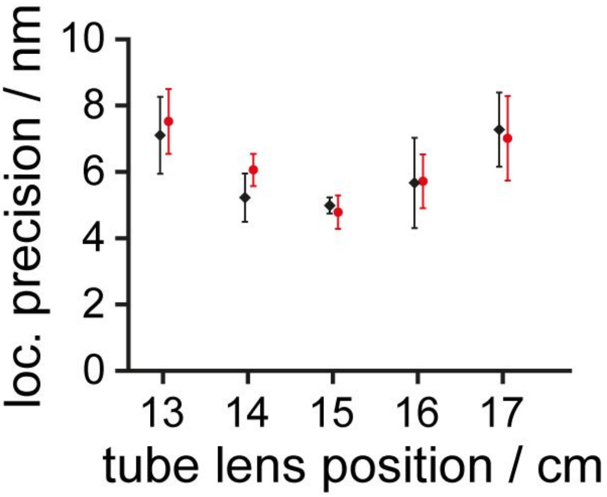

Figure S1: Tube lens positioning. Black: x. Red: y. Experimental localization precision of fluorescent beads imaged at several tube lens positions.

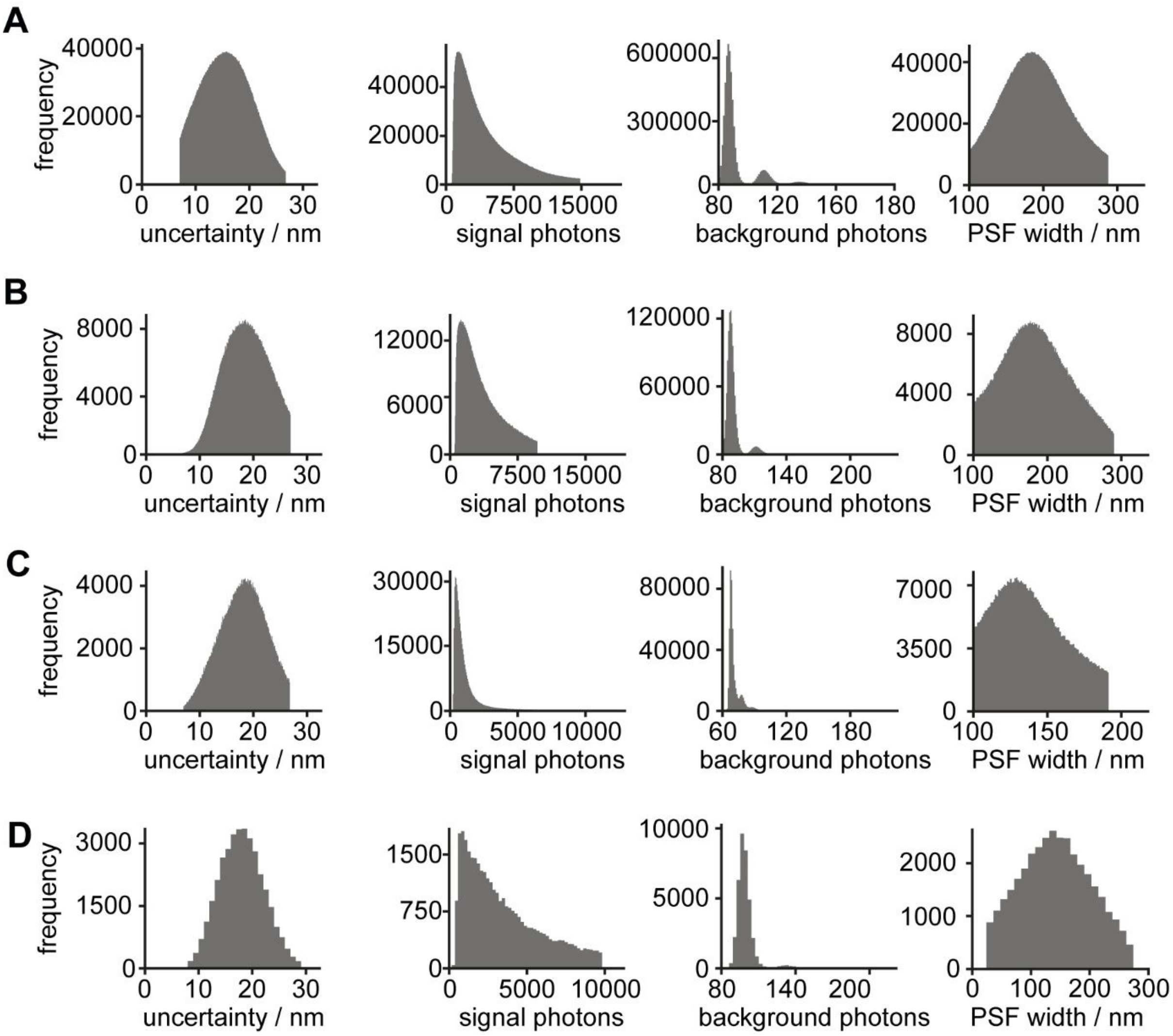

Figure S2: Histograms of localization precisions, signal and background photons, and PSF widths. A) One-color imaging of microtubules, AF647. B) Two-color imaging of microtubules, AF647. C) Two-color imaging of microtubules, CF568. D) One-color imaging of the glycocalyx, AF647.

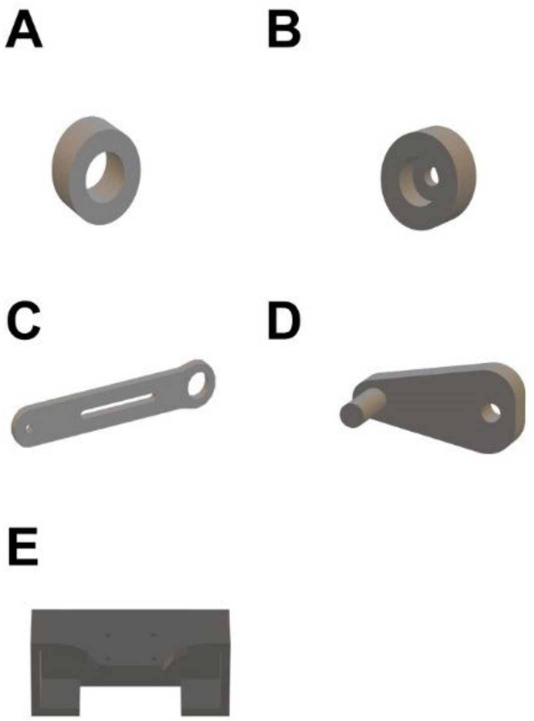

Figure S3: Custom parts. Dimensions: A) Wheel; outer Ø = 6 mm, inner Ø = 3.5 mm, thickness = 3 mm; B) Connector lock, outer Ø = 5 mm, inner Ø = 2.5 mm, hole Ø = 1mm, thickness = 3mm; C) Lever, length=15 cm, width=15-18 mm (lower to higher edge), thickness = 4 mm, D) Lever-motor-connector, width = 10 mm, length = 15 mm, thickness = 4 mm. E) Sample stage. Height = 3.5 cm, length = 9 cm, width = 3 cm. Parts A)-D) are 3D printed, part E) is engineered.

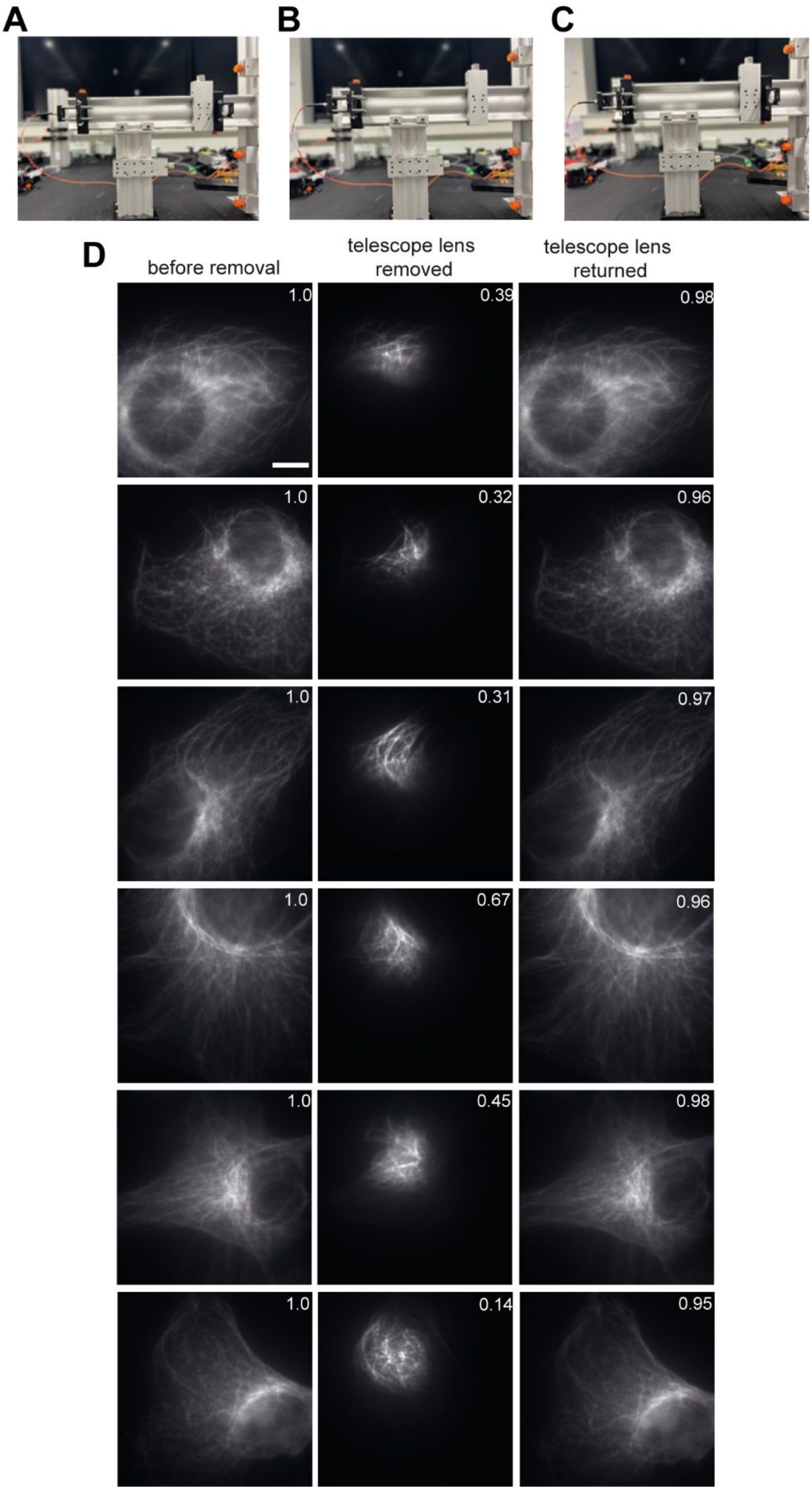

Figure S4: Setup robustness inspection via lens configuration alteration. A) Initial configuration. B) Telescope lens removed. C) Telescope lens returned. D) Additional data corresponding to main text Figure 2A. Pearson correlation coefficients are given at the top right of each image with respect to the image before lens removal. Scale bar: 10 μm.

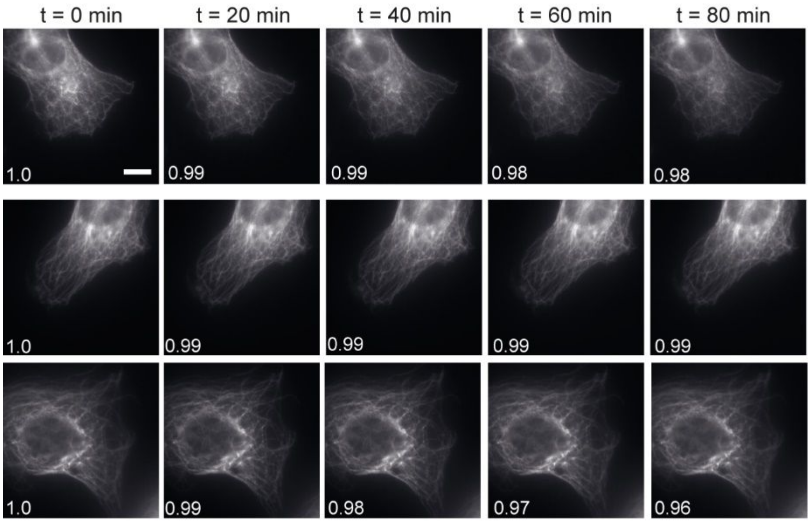

Figure S5: Mechanical stability of the setup over 80 minutes. Data corresponding to main text Figure 2B. Pearson correlation coefficient are given at the bottom left of each image with respect to the image at t=0 min. Scale bar: 10 µm.
